# Feature enhancement and fusion-optimized defect detection model for Sanhua plums

**DOI:** 10.3389/fpls.2026.1759809

**Published:** 2026-01-30

**Authors:** Yu Chen, Yuanxia Zhang

**Affiliations:** Yulin Normal University, Yulin, China

**Keywords:** defective fruit detection, lightweight model, multi-weather simulation, Sanhua plum, small object detection, YOLO-CMA

## Abstract

To achieve precise and efficient detection of abnormal Sanhua plums, this study first constructed a specialized image dataset encompassing five categories: diseased fruit, insect-damaged fruit, bird-pecked fruit, cracked fruit, and normal fruit. To mitigate the initial class imbalance, a multi-weather simulation data augmentation strategy was employed, which expanded the dataset to 10,000 images and achieved a balanced distribution. After systematically evaluating multiple state-of-the-art detection models, YOLOv12 was selected as the baseline model due to its high recall and extreme lightweight nature. To overcome the core challenge of detecting minute defects like insect-damaged fruit, this study innovatively proposed the YOLO-CMA model. This model integrates the C2fCIB module to enhance small-object feature extraction capabilities and incorporates the C3k2_Mambaout module to optimize the discriminative fusion of multi-scale features. Ablation experiments demonstrated that the synergistic operation of C2fCIB and C3k2_Mambaout improved detection performance. For insect-damaged fruit detection, mAP50 and precision were boosted by 2.7% and 5.8%, respectively, compared to the baseline model, reaching 0.639 and 0.91. When compared to other YOLO variants, YOLO-CMA achieved the lowest computational cost (5.9 GFLOPs) and parameter count (2.43 M) while maintaining competitive detection precision, demonstrating significant edge deployment advantages. This study provided a comprehensive technical solution—from data construction to model optimization—for addressing the challenge of detecting minute defects in agricultural products, offering substantial practical value and application potential.

## 1 Introduction

Sanhua plum is a stone fruit widely cultivated in western Guangdong, China. Its fruit is highly valued for its vibrant color, unique sweet-tart flavor, and rich nutritional content—particularly vitamin C—earning it significant commercial value and market recognition. Automated post-harvest quality inspection is crucial for enhancing industrial efficiency. However, detecting anomalies proves challenging due to the diverse types and distinct characteristics of defects, particularly minute imperfections like insect-bored holes (≤2-mm diameter) and bird-pecked notches, which are extremely difficult to identify in complex orchard environments (Wu et al., 2024; Tang et al., 2025). These small targets pose significant challenges, including weak feature representation, variable morphology, and a high tendency to be confused with natural fruit surface textures (Chutichaimaytar et al., 2025; Ren et al., 2025). Concurrently, post-harvest processing equipment for Sanhua plums urgently requires lightweight models capable of real-time deployment on embedded edge devices (Kim et al., 2025; Qiu et al., 2025). Furthermore, class imbalance in the dataset—such as sparse samples of insect-damaged or bird-pecked fruits—severely hampers balanced model performance improvement (Pazhanivelan et al., 2025). Therefore, this study clearly focused on the following: optimizing small object detection performance, addressing class imbalance through data augmentation, and designing detection models that balance high precision with extreme lightweighting to tackle the practical challenges of automated post-harvest sorting for Sanhua plums.

In recent years, deep learning-based object detection models have gained widespread application in agriculture (Nkonjoh et al., 2025). Among these, the YOLO series models have become a research hotspot due to their outstanding real-time performance (Sonawane and Patil, 2025). Researchers have explored YOLOv5 (Nguyen et al., 2025a; Ali et al., 2025), YOLOv7 (Sun et al., 2024), YOLOv8 (Shen et al., 2025; Zhou et al., 2024), YOLOv10 (Yuan et al., 2025), and the latest YOLOv12 (Kim et al., 2025) across diverse agricultural scenarios. Concurrently, Transformer-based architectures like RT-DETR have demonstrated strong potential, achieving lightweight and high-precision improvements in disease detection for crops such as tomatoes (Zhao Z. et al., 2024), corn (Guo et al., 2025), and apples (Wang et al., 2025b). However, the aforementioned studies primarily focused on relatively conspicuous diseases, medium-sized targets, or general agricultural scenarios, while research on detecting minute, early-stage defects on fruit surfaces (e.g., early insect holes and fine cracks) remains relatively limited.

To tackle the challenge of small object detection, multi-scale feature fusion and attention mechanisms are two widely validated, effective strategies. The YOLO-LF model proposed by Wang et al. significantly enhances the detection capability of small disease targets by strengthening multi-scale information interaction (Wang et al., 2025a). Studies like those on SAW-YOLO (Wu et al., 2024) and CTB-YOLO (Chutichaimaytar et al., 2025) further specialized model architecture optimizations for specific agricultural small targets. Regarding attention mechanisms, the collaborative attention and self-knowledge distillation mechanism adopted by Han et al. (2026), along with the hybrid attention mechanism integrated into Faster R-CNN by Guan et al. (2025), both effectively enhance the model’s feature discrimination capability in complex backgrounds. The prototype attention network proposed by Fu et al. offers novel insights for refining feature representations (Fu and Shi, 2025). Furthermore, explorations of new technologies like Vision Transformers (Scutelnic et al., 2026) and novel activation functions (Nguyen et al., 2025b) have injected vitality into model evolution. Although these methods improve general small object detection, for fruits with complex color and texture like Sanhua plum, existing models still struggle to achieve highly discriminative feature extraction and retention under lightweight constraints.

Regarding model lightweighting and edge deployment, researchers have conducted extensive practical work. The study of Feng et al. on YOLO-Citrus (Feng et al., 2025) and that of Gao et al. on SCS-YOLO (Gao et al., 2025) both achieved a balance between precision and speed in complex environments. A series of lightweight adaptations to RT-DETR (Kong et al., 2025; Liu et al., 2025; Cheng et al., 2025) and the mobile-optimized NanoDet-Plus (Qiu et al., 2025) collectively expand the technical options for lightweight models. Research by Kang et al. (2024) and Pan et al. (2025) respectively demonstrated specific pathways for achieving lightweight solutions in tomato growth monitoring and wheat lodging detection. However, these lightweight designs often sacrifice feature representation capability, and maintaining sensitivity to millimeter-scale defects under extreme lightweight conditions remains a major challenge.

Data augmentation and robustness enhancement are crucial for addressing real-world complexity. Scutelnic et al. emphasized the importance of handling environmental diversity through multi-model integration and multispectral analysis (Scutelnic et al., 2026). Sun et al., focusing on stored grain pests, demonstrated the potential of lightweight models for small object detection (Sun et al., 2026). These studies, alongside multi-class detection practices (Pazhanivelan et al., 2025), underscore the critical importance of high-quality data and robust models. However, systematic research on multi-weather, multi-environment simulation data augmentation for Sanhua plum has not been conducted, lacking specialized augmentation schemes that can simultaneously address class imbalance and complex environmental variations.

Despite these substantial achievements, addressing the specific challenge of detecting multi-class anomalies in Sanhua plums presents the following challenges.

1. Insufficient extraction and retention of minute defect features: General models exhibit limited capability in extracting shallow-level detail features of targets with extremely low pixel coverage (e.g., insect holes) and retaining these features within deep networks.
2. Enhanced model robustness under variable conditions: Natural lighting and weather variations (e.g., rain and fog) pose significant challenges to model generalization capabilities.
3. Insufficient synergistic optimization of precision and lightweight design: Maintaining or further reducing computational overhead while incorporating enhancement modules remains a major challenge in resource-constrained edge computing environments.
4. Lack of specialized solutions: There is a shortage of dedicated datasets for multi-class anomaly detection in Sanhua plum fruits (especially five-class classification, including normal fruits) and systematic optimization approaches from data to model.

Therefore, this study targeted a more focused challenge: achieving robust detection of microscopic defects (e.g., ≤2-mm insect holes) in Sanhua plums against complex textures, under extreme lightweight constraints. We proposed a “Collaborative-Decoupled” optimization paradigm. Our contributions are as follows.

1. Enhanced specialized dataset: A five-category dataset with a multi-weather simulation augmentation strategy to boost environmental robustness.
2. High-recall, ultra-light baseline: YOLOv12 is selected as the baseline, reserving parameter budget for precision optimization.
3. Innovative YOLO-CMA model and core design: The core innovation is the C2fCIB and C3k2_Mambaout modules. They decouple and tackle two key bottlenecks: “semantic retention of minute features in deep networks” and “discriminative fusion of multi-scale features”. Their synergistic operation yields a 2.7% mAP50 and 5.8% accuracy gain for insect-damaged fruit, while achieving the lowest computational cost and parameters among all compared YOLO variants (5.9 GFLOPs, 2.43 M).
4. Comprehensive validation: Ablation and comparative experiments validate the solution, providing a complete technical pipeline from data to model.

## 2 Construction of Sanhua plum abnormal fruit dataset

### 2.1 Data collection and annotation specifications

**Figure 1:**
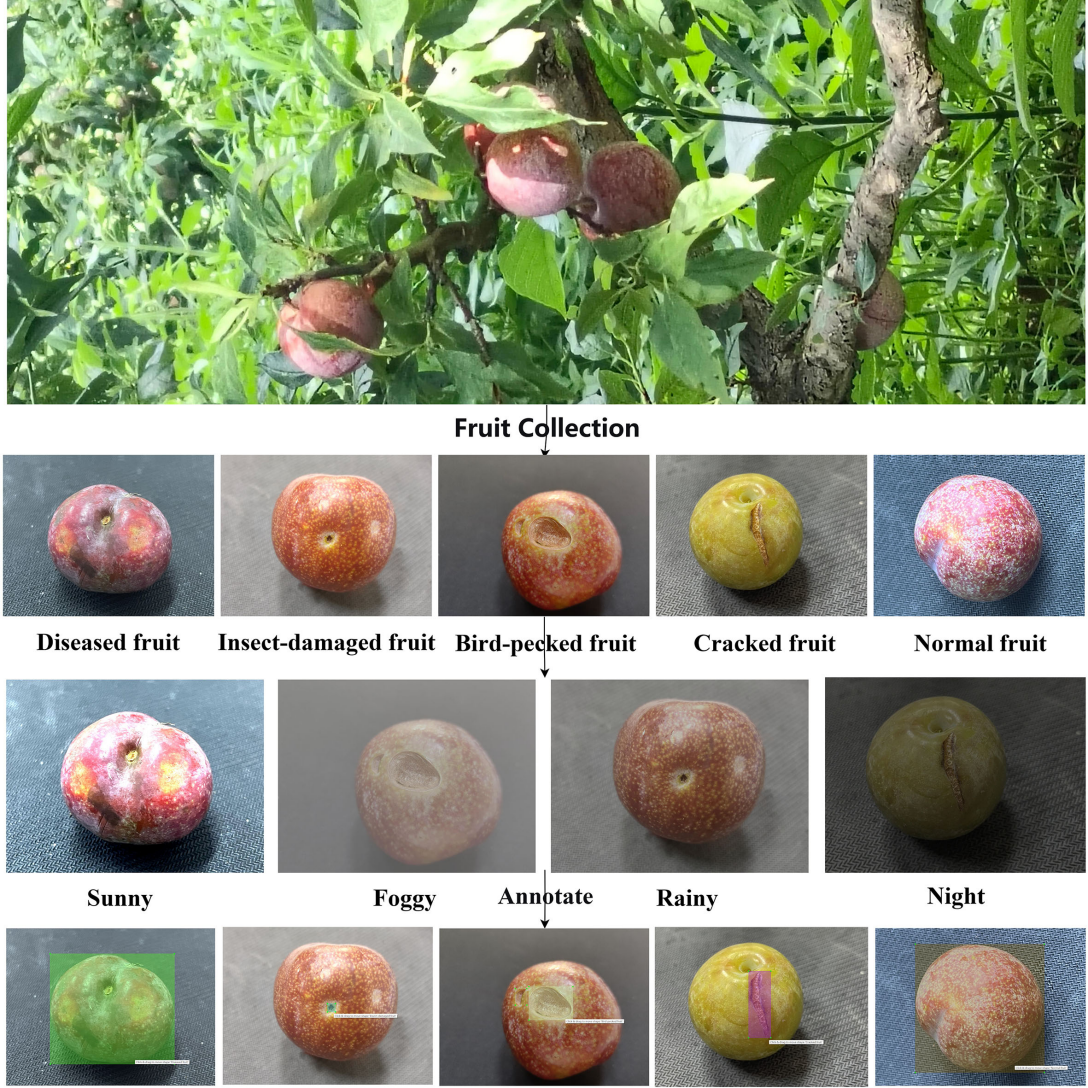
The complete image data processing workflow of Sanhua plums from raw collection to a balanced annotated dataset.

Data collection was conducted during the 2024–2025 Sanhua plum harvest season in the core production area of Xinyi, Maoming, Guangdong Province. Multiple mainstream smartphones were used as capture devices, all set to automatic shooting mode to ensure high-quality images under natural lighting conditions. The initial collection yielded 5,915 valid images, with the initial distribution across categories shown in the upper half of **Figure 1**. The specific distribution was as follows: 1,767 diseased fruits, 788 insect-damaged fruits, 709 bird-pecked fruits, 1,524 cracked fruits, and 1,127 normal fruits. This distribution revealed a significant imbalance, with notably insufficient samples for the two critical defect categories: insect damage and bird pecking.

To meet the high-quality data requirements for abnormal fruit detection in Sanhua plums, this study systematically constructed a specialized image dataset covering five defect categories (diseased, insect-damaged, bird-pecked, cracked, and normal fruits). The dataset construction followed a standardized workflow, as illustrated in **Figure 1**, encompassing the entire process from raw data collection to final dataset annotation.

Professional annotation tools were annotated meticulously using professional annotation tools, as depicted in the “Annotate” stage of **Figure 1**. Uniform annotation standards were established: diseased fruits were labeled as whole fruit sections, insect-damaged fruits focused on minute insect holes (≤2-mm diameter), bird-pecked fruits accurately marked areas with surface notches, cracked fruits were finely annotated with epidermal fissures, and normal fruits were labeled as intact, defect-free surfaces. All annotations underwent verification by an agricultural expert team to ensure consistency and precision.

### 2.2 Data augmentation based on multi-weather simulation

To address sample imbalance and enhance model environmental adaptability, this study designed a data augmentation scheme based on multi-weather simulation. Enhancement parameter configurations are detailed in **Table 1**: “↑”, enhancement parameter (value > 1.0); “↓”, weakening parameter (value < 1.0); “Value Range”, the random sampling range for the parameter; and “-”, the parameter was not used for that weather type. This scheme simulates four typical weather conditions.

**Table 1:** Parameter configuration for multi-weather simulation data augmentation for environmental robustness.

Weather type	Brightness adjustment	Contrast adjustment	Saturation adjustment	Gaussian blur	Raindrop effect	Fog effect	Noise effect
Sunny	1.1–1.5↑	1.1–1.4↑	1.1–1.5↑	–	–	–	–
Rainy	0.6–0.9↓	–	0.7–0.9↓	0.5–1.5↓	Number of raindrops: 30–100 Raindrop length: 10–25 px Raindrop width: 1–2 px	–	–
Foggy	–	0.6–0.8↓	0.7–0.9↓	0.8–2.0↓	–	Fog color: 180–230 Fog intensity: 0.2–0.5	–
Night	0.3–0.6↓	–	0.5–0.8↓	–	–	–	Noise intensity: 5–15

1. Clear weather: Enhances brightness (1.1–1.5), contrast (1.1–1.4), and saturation (1.1–1.5) to accentuate surface texture details.
2. Rainy conditions: Reduces brightness (0.6–0.9) and saturation (0.7–0.9), applies Gaussian blur (0.5–1.5), and simulates raindrops.
3. Haze/smog conditions: Reduce contrast (0.6–0.8) and saturation (0.7–0.9), combined with Gaussian blur (0.8–2.0) and simulated fog effects.
4. Night conditions: Significantly reduce brightness (0.3–0.6) and saturation (0.5–0.8) and add random noise (intensity 5–15).

**Figure 2:**
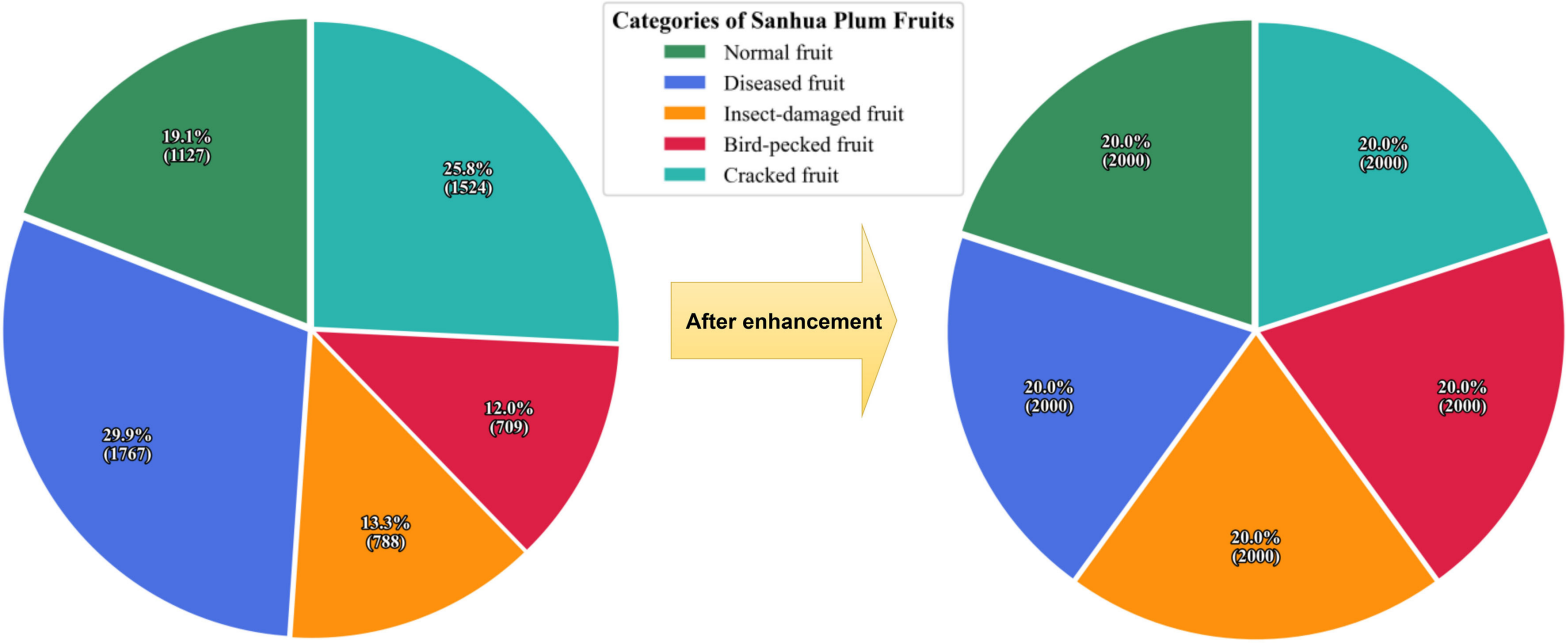
Comparison of sample distributions for each category before and after data augmentation based on multi-weather simulation.

Through targeted multi-weather simulation enhancement, each sample category was expanded to 2,000 images, bringing the total dataset size to 10,000 images. The data distribution before and after augmentation is shown in **Figure 2**, demonstrating an ideal equilibrium across the five sample categories. The final dataset was partitioned into training (7,000 images), validation (2,000 images), and test (1,000 images) sets at a 7:2:1 ratio, providing comprehensive data support for model training and evaluation.

It is important to note that while this strategy offers advantages in balancing the dataset and enhancing environmental diversity, the standalone experiment in Section 5.2 reveals that its quantitative impact on the absolute performance of the chosen baseline model (YOLOv12) on the standard test set is limited. Therefore, the primary contribution of this strategy lies in improving model robustness and generalization potential, rather than directly and significantly boosting baseline accuracy. Subsequent discussions regarding the augmentation effect will be framed objectively based on this understanding.

## 3 Baseline model selection and performance bottleneck analysis

### 3.1 Multi-model evaluation and baseline model establishment

To establish the most suitable baseline model for the edge-based anomaly detection task of Sanhua plum, this study evaluated five object detection models—YOLOv12 (Tian et al., 2025), RT-DETR (Zhao Y. et al., 2024), Hyper-YOLO (Feng et al., 2024), NanoDet (Van Thanh et al., 2025), and SSD (Liu et al., 2016). The quantitative metrics of each model on the test set are shown in **Table 2**, providing objective evidence for model selection.

**Table 2:** Comprehensive performance comparison of different object detection models on the Sanhua plum defective fruit dataset.

Model	Precision	Recall	F1-Score	mAP50	Model size (M)
YOLOv12	0.934	0.889	0.905	0.909	5.17
RT-DETR	0.948	0.904	0.923	0.929	16.2
Hyper-YOLO	0.967	0.90	0.927	0.924	7.24
NanoDet	0.989	0.866	0.908	0.817	16.3
SSD	0.986	0.699	0.754	0.862	92.7

**Table 2** reveals significant variations across metrics among different models. Hyper-YOLO demonstrates superior performance in precision (0.967) and F1-Score (0.927), highlighting its balanced advantage in both precision and recall. RT-DETR slightly outperforms in mAP50 (0.929) and recall (0.904), indicating more balanced detection performance across different categories. However, these performance advantages come at the cost of significantly increased model complexity.

**Figure 3:**
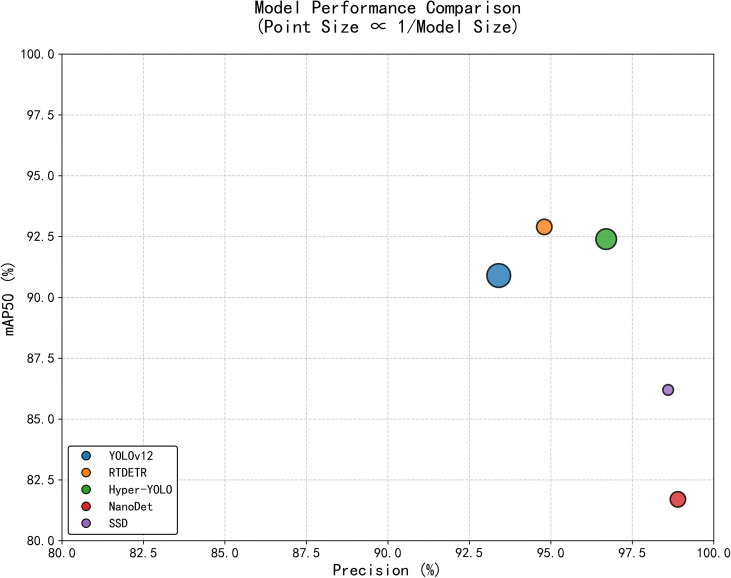
Precision–lightweight trade-off analysis of different detection models (point size is inversely proportional to model size).

To visually illustrate the trade-off between precision and lightweight design across models, this study plotted a precision–performance distribution scatter plot (**Figure 3**). Precision is plotted on the x-axis and mAP50 on the y-axis, with model size represented by scatter point area (larger areas indicate lighter models). The distribution pattern clearly reveals the following: Hyper-YOLO, RT-DETR, and NanoDet cluster in the high-precision region on the right side of the chart, but with smaller scatter areas (indicating larger model sizes); SSD exhibits high precision but mediocre mAP50 performance alongside the largest model size; YOLOv12 uniquely occupies the upper-left quadrant, forming a distinct Pareto optimal point—achieving lightweighting with the largest scatter area (minimum model size of 5.17M) while maintaining excellent mAP50 (0.909).

A deeper analysis of YOLOv12’s metrics reveals its core advantages in three areas: first, in model lightweighting, its 5.17MB size represents only 31.9% of RT-DETR and 31.7% of NanoDet, making it highly valuable for edge deployment. Second, its recall rate of 0.889 is the highest among all models, significantly reducing defect fruit miss rates and ensuring sorting quality. Finally, its F1-Score of 0.905 demonstrates a well-balanced trade-off between precision and recall—critical for practical applications requiring both false-positive control and false-negative prevention.

Notably, the current model comparison also reveals room for improvement in YOLOv12. Its precision (0.934) is relatively low, particularly when compared to NanoDet (0.989), where the gap reaches 5.5 percentage points. This reflects existing models’ limitations in complex background discrimination and subtle feature differentiation. This phenomenon may stem from the model’s limited ability to characterize minor defects during feature extraction or insufficient discrimination of similar features at the decision-making level.

Based on the above analysis, this study selected YOLOv12 as the baseline model for subsequent improvements. This decision is based on the following considerations: first, its extreme lightweight nature reserves ample parameter space for introducing improvement modules later. Second, its high recall rate ensures comprehensive defect coverage, providing a solid foundation for subsequent precision optimization. Third, as the latest evolution in the YOLO series, its architecture integrates cutting-edge object detection concepts and offers excellent scalability.

Subsequent research will focus on enhancing the model’s feature discrimination capabilities. This will be achieved by introducing attention mechanisms to strengthen the capture of small object features and optimizing feature fusion pathways to enhance the integration of semantic information and detailed features. While maintaining the existing lightweight advantages and high recall rate, efforts will concentrate on improving the model’s precision rate, ultimately achieving a comprehensive enhancement in the overall performance of Sanhua plum fruit anomaly detection.

### 3.2 In-depth analysis of baseline model performance bottlenecks

To develop a high-performance Sanhua plum anomaly detection model, this study first trained and evaluated the YOLOv12 baseline model to systematically analyze its performance across five fruit detection tasks. Detailed performance metrics for each fruit category are presented in **Table 3**.

**Table 3:** Detailed performance metrics of the baseline model (YOLOv12) on five types of Sanhua plum fruits.

Category	Precision	Recall	mAP50	mAP50–95
Diseased fruit	0.969	0.998	0.994	0.974
Insect-damaged fruit	0.852	0.532	0.612	0.34
Bird-pecked fruit	0.939	0.921	0.949	0.677
Cracked fruit	0.94	0.976	0.988	0.714
Normal fruit	0.979	0.99	0.994	0.946

Through in-depth analysis of the data in **Table 3**, two critical issues with the baseline model can be clearly identified.

1. Severe inadequacy in detecting small target defects: As a typical micro-defect (wormhole diameter ≤2 mm), worm-damaged fruit exhibits significantly lower recall (0.532) and mAP50 (0.612) compared to other categories. This indicates that nearly half of the worm-damaged fruits remain undetected, exposing the model’s pronounced deficiency in perceiving small-scale targets.
2. Urgent need to enhance model classification precision: The 0.852 precision rate for detected insect-damaged fruit indicates a substantial proportion of misclassifications even among detected cases. The model struggles to accurately distinguish insect holes from similar features like natural fruit surface textures and spots.

These bottlenecks primarily stem from limitations in the baseline model’s feature extraction and fusion mechanisms: small object features in deep networks are easily diluted, and existing feature aggregation strategies lack sufficient discrimination capability for minute defects.

## 4 Improved YOLO-CMA model design

### 4.1 Overall architecture overview

**Figure 4:**
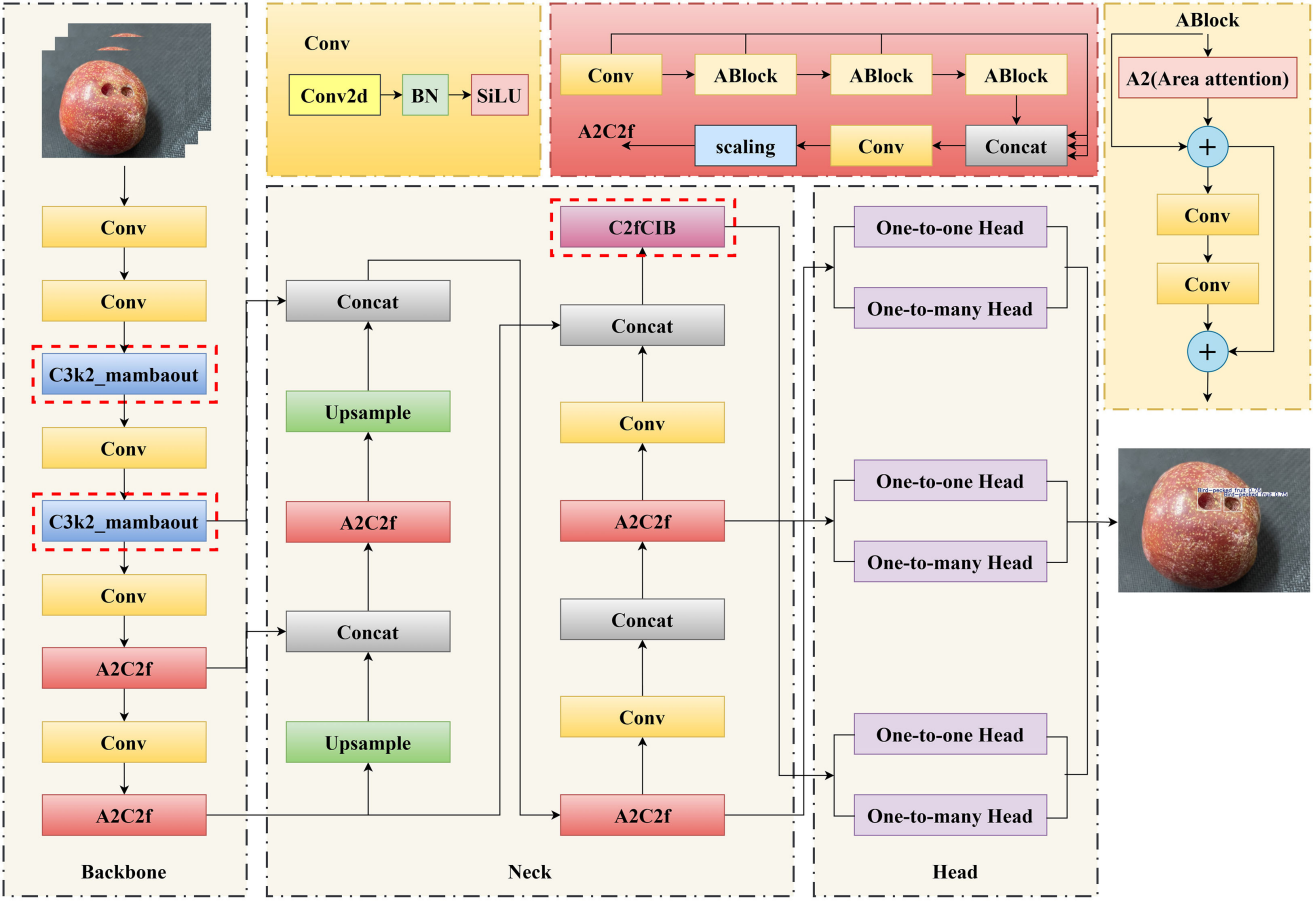
Overall architecture of the YOLO-CMA model, highlighting the positions of the C2fCIB and C3k2_Mambaout modules in the backbone and neck networks.

To tackle the challenge of detecting anomalies in Sanhua plums, particularly minute defects like insect-damaged fruit, this study proposed an improved object detection model named YOLO-CMA. Based on systematic evaluation and bottleneck analysis, it adopted YOLOv12 as the baseline for its high recall and extreme lightweight characteristics. The core innovation lies in enhancing feature extraction by injecting the C3k2_Mambaout module into the deep layers of its backbone network, and optimizing multi-scale feature fusion by embedding the C2fCIB module into the critical paths of its neck network. This constructs a comprehensive solution dedicated to the precise identification of minute defects in Sanhua plums within complex agricultural scenarios. The overall architecture of the YOLO-CMA model features a logically coherent design, with its complete data processing flow from input to output illustrated in **Figure 4**.

As illustrated in **Figure 4**, the YOLO-CMA architecture can be distinctly divided into three functionally distinct yet closely integrated components: the backbone network, the neck network, and the detection head. Their collaborative mechanism operates as follows.

1. Backbone: deep feature extraction and enhancement

The backbone network serves as the model’s foundation, performing hierarchical feature extraction from raw input images to generate a series of feature maps with progressively deeper levels and increasing receptive fields. YOLO-CMA retains the efficient four-stage pyramid structure of baseline models (e.g., CSPNet design), outputting feature maps at 1/8, 1/16, and 1/32 downsampling rates (P3, P4, and P5, respectively). The core innovation of this study lies in introducing the C3k2_Mambaout module at the P5/32-large layer, which handles the deepest features with the largest receptive field. Based on advanced gated convolution principles, this module employs a unique dual-path parallel processing mechanism (one path focuses on deep feature extraction, while the other path enhances features through a gating mechanism). This significantly strengthens the network’s ability to capture and preserve minute, subtle features (such as insect-bored holes), effectively mitigating the core issue of semantic information for small objects being drowned out or diluted in deep networks. It provides more discriminative foundational feature representations for subsequent processing stages.

2. Neck network: multi-scale feature fusion and refinement

The neck network serves as an “information hub”, tasked with effectively aggregating and refining feature maps from different semantic levels output by the backbone network. YOLO-CMA adopts a widely validated bidirectional feature pyramid network (e.g., PANet) as its fundamental framework, enabling top-down and bottom-up bidirectional feature fusion. A key innovation in this study is the replacement of some original units within this fusion pipeline with the newly designed C2fCIB module. The C2fCIB module integrates a compact inverse residual block, employing a multi-branch architecture with depthwise separable convolutions. This achieves efficient fusion and enhancement of shallow spatial details (e.g., insect hole edges and crack textures) with deep semantic information (e.g., fruit category) at minimal computational cost. This design ensures that feature maps fed to the detection head are rich in both detail and semantic information, particularly enhancing feature discriminability for small-scale defect targets.

3. Detection head: object classification and precise localization

The detection head serves as the model’s decision terminal, receiving the optimized multi-scale fused feature maps from the neck network and concurrently executing two core tasks: object category classification and bounding box regression. YOLO-CMA adopts the mainstream decoupled detection head design, separating classification and regression tasks. Two independent lightweight neural networks handle these tasks, effectively avoiding learning conflicts between the two objectives and thereby improving model convergence and final precision. Furthermore, addressing the extreme size variation in the Sanhua plum dataset (ranging from whole fruits to millimeter-scale insect holes), we performed targeted re-clustering and optimization of anchor ratios and sizes for the three detection heads (corresponding to P3, P4, and P5 feature maps). This ensures that objects of different scales are captured by the most suitable detection scale, enabling comprehensive and precise detection from macro-level diseased fruits to micro-level insect holes.

Collaborative mechanism summary: The YOLO-CMA model forms an efficient detection pipeline through the intricate coordination of these three components. Input images first undergo deep feature enhancement via the backbone network integrated with the C3k2_Mambaout module. Subsequently, these features undergo comprehensive multi-scale fusion and refinement within the neck network integrated with the C2fCIB module. Finally, the optimized features are fed into the decoupled detection head to complete high-precision classification and localization. The C3k2_Mambaout and C2fCIB modules play pivotal roles in the “depth” of feature extraction and the “breadth” of feature fusion, respectively. Their synergistic operation is the fundamental reason that the model achieves significant performance improvements in detecting small-object defects while maintaining lightweight characteristics.

To clearly articulate the design motivation and innovations of the core modules in YOLO-CMA, and to illustrate their fundamental differences from existing mainstream lightweight modules, a systematic comparison of the structures and functions of relevant modules is conducted. As shown in **Table 4**, while classical modules like C3 and C2f can achieve basic feature extraction and fusion, they are not designed for minute defect detection, and the semantic information of small objects is easily lost in deep networks. General lightweight modules [e.g., Compact Inverted Bottleneck (CIB)] focus on compressing parameters but often at the cost of feature representation capability. To address these limitations, the proposed C2fCIB and C3k2_Mambaout modules introduce targeted optimizations: C2fCIB enhances the extraction and gradient flow of small-object features while maintaining lightness by incorporating a compact inverted bottleneck block; C3k2_Mambaout optimizes the discriminative fusion of multi-scale features using a dual-path gating mechanism. These two innovations are the key to solving the bottleneck in detecting minute defects in Sanhua plums.

**Table 4:** Structural comparison of lightweight channel-mixing modules.

Module name	Core structure	Main function	Applicable scenario	Problems addressed in this paper
C3 (YOLOv5)	3 conv layers + residual connection	Feature extraction and channel fusion	General object detection	Not optimized for minute defects; small-object features easily diluted in deep networks
C2f (YOLOv8)	Multi-branch feature concatenation	Enhance feature diversity	Medium-scale object detection	Lacks deep semantic retention and focus capability for small-object features
CIB (lightweight)	Depthwise separable conv + inverted bottleneck	Reduce parameters, improve efficiency	Mobile deployment	Weak feature expression, prone to loss of minute details
C2fCIB (ours)	C2f framework + compact inverted bottleneck block	Enhance small-object feature extraction and gradient flow	Minute defect detection	Solves the dilution and propagation challenges of small-object features in deep networks
C3k2_Mambaout (ours)	Dual-path gated convolution + feature enhancement	Optimize multi-scale feature fusion and discriminability	Small object recognition in complex backgrounds	Improves model’s discrimination of subtle defects, significantly reducing false positives

### 4.2 C2fCIB module—enhanced feature extraction and gradient flow

In object detection models, the design of feature fusion modules is crucial for effectively transmitting and integrating multi-scale information. To further enhance the feature fusion and representation capabilities for minute defects (e.g., insect holes) in Sanhua plums beyond the YOLOv12 baseline model, while maintaining computational efficiency on edge devices, this study introduced the C2fCIB (C2f with Compact Inverted Bottleneck) module and applied it to key layers (e.g., P5/32-large layer) to replace the original structure (Wang et al., 2024).

**Figure 5:**
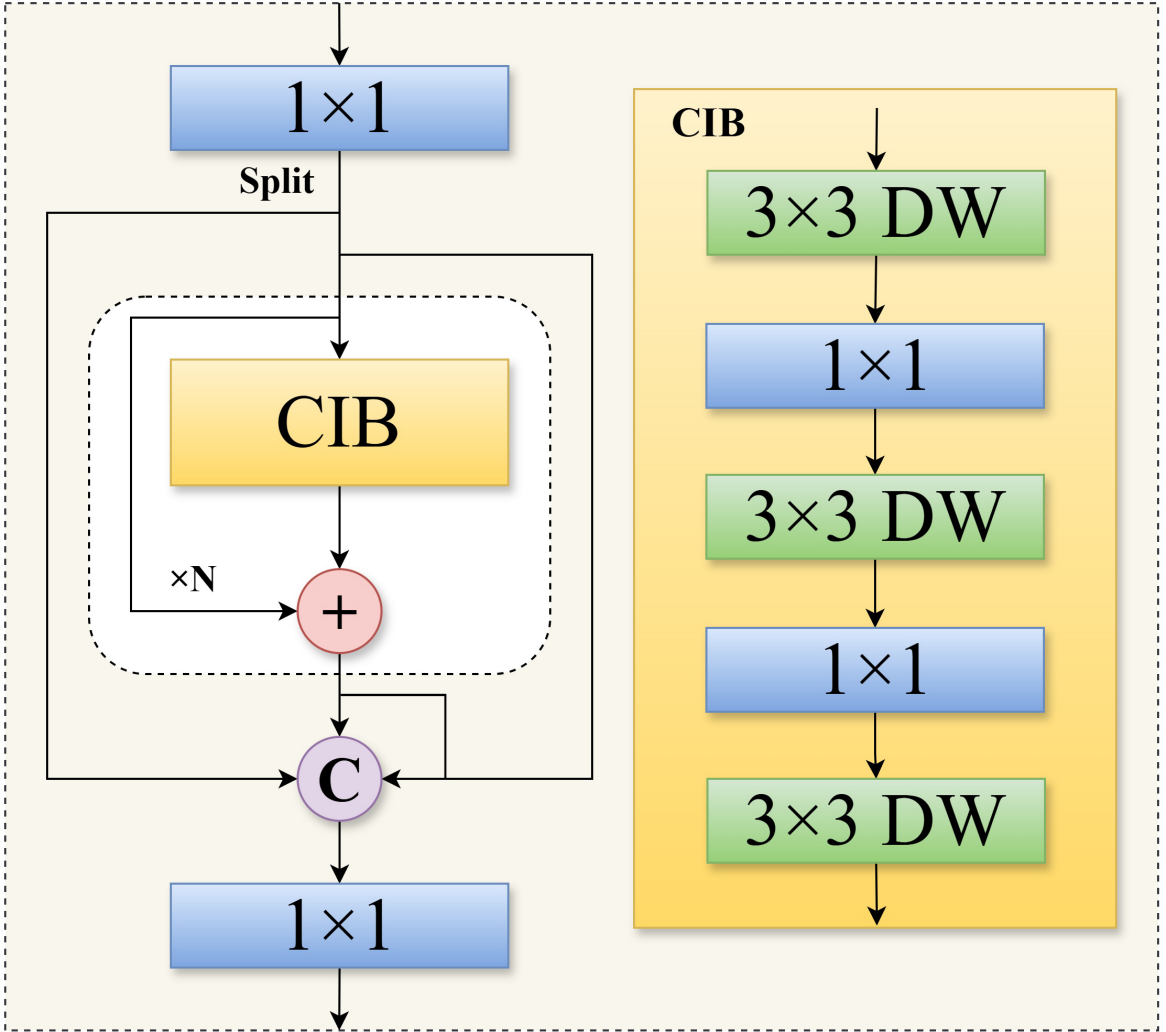
C2fCIB structure diagram.

The core of the C2fCIB module lies in embedding the CIB block as a fundamental building unit within the C2f multi-branch fusion framework. This architecture aims to optimize the feature transformation process through CIB blocks while enriching the feature flow within the multi-path fusion framework. This dual approach enhances the model’s ability to capture small-object features while controlling computational overhead. The C2fCIB block structure draws inspiration from deep separable convolutions and inverted bottleneck connections to construct an efficient feature transformation unit. Its architecture is illustrated in **Figure 5**.

The data processing flow within this block is as follows.

- Channel expansion (expand): Input feature maps first undergo channel dimension expansion via a 1 × 1 pointwise convolution layer, mapping features to a higher-dimensional space to enhance representational capacity.
- Depthwise feature extraction: A 3 × 3 depthwise convolution performs spatial filtering on the expanded feature map. This operation effectively captures spatial contextual information while significantly reducing the number of parameters.
- Channel squeeze and activation: A 1 × 1 dot convolution reduces the number of feature channels back to the target dimension, enabling cross-channel information fusion. The SiLU activation function is introduced to enhance non-linear expressive power.

Within the C2fCIB module, multiple serial CIB blocks form the core feature processing pipeline. The workflow proceeds as follows: input features are split into two branches after an initial convolutional layer. One branch serves as a shortcut connection, while the other sequentially passes through multiple CIB blocks for feature refinement. Finally, outputs from all branches are concatenated along the channel dimension and undergo feature fusion via a terminal convolutional layer.

This architecture offers dual advantages: the multi-branch structure and shortcut connection ensure smooth gradient flow, facilitating model training; the cascaded CIB blocks and feature concatenation enable the module to output composite features integrating diverse receptive fields and abstraction levels, enhancing multi-scale defect detection capabilities (Wang et al., 2024).

The computational efficiency of the C2fCIB module primarily stems from its adoption of a deep separable convolution structure. For an input feature map of size H×W×C, the computational complexity of standard convolution $R_{1}$ can be expressed as follows:

(1) $$\begin{array}{c} {\mathbb{R}}_{1}=\text{O}(\frac{9}{2}HWC^{2}) \end{array}$$

In contrast, the separable convolution in CIB decomposes the computation into two steps, with a total complexity of $R_{2}$:

(2) $$\begin{array}{c} {\mathbb{R}}_{2}=\text{O}(2HWC^{2}+\frac{9}{2}HWC\;) \end{array}$$

Comparing the two, the deep separable convolution shifts the dominant computational complexity from $K^{2}\cdot C^{2}$ in standard convolution to $C^{2}+K^{2}\cdot C\;$. Since the number of channels C is typically large, this structural change significantly enhances computational efficiency. Consequently, the C2fCIB module enhances feature expression capabilities while keeping computational overhead within reasonable bounds.

In summary, the C2fCIB module achieves a balance between feature extraction capability and computational efficiency by integrating the efficient CIB block into a multi-path fusion framework. Subsequent ablation experiments (see **Table 5**) demonstrated that this module plays a crucial role in enhancing the detection performance of small-scale defect targets such as worm-damaged fruits, with notable improvements observed in both recall and mAP metrics.

**Table 5:** Hyperparameter configuration for the model training phase.

Parameter category	Parameter name	Setting value
Baseline training setup	Training epochs	200
	Batch size	32
	Input image size (imgsz)	416 × 416
	Warmup epochs	3
Optimizer settings	Optimizer	Auto (adaptive selection of SGD/AdamW)
	Initial learning rate (lr0)	0.01
	Momentum	0.937
	Weight decay	0.0005
Loss function settings	Bounding box loss (box)	7.5
	Classification loss (cls)	0.5
	Distribution focal loss (dfl)	1.5
Data augmentation strategy	Random horizontal flip probability (flipud)	0.5 (probability)
	HSV augmentation (hsv_h/hsv_s/hsv_v)	0.015/0.7/0.4
	Translate	0.1
	Scale	0.5
	Mosaic augmentation (mosaic)	1.0 (probability)
	Auto augment (auto_augment)	randaugment
Hardware and training configuration	Device	NVIDIA GeForce RTX 3060
	Data loading workers	8
	Mixed precision training (amp)	Yes
	Early stopping patience (patience)	100

### 4.3 C3k2_Mambaout module—optimizing feature fusion and discrimination capabilities

**Figure 6:**
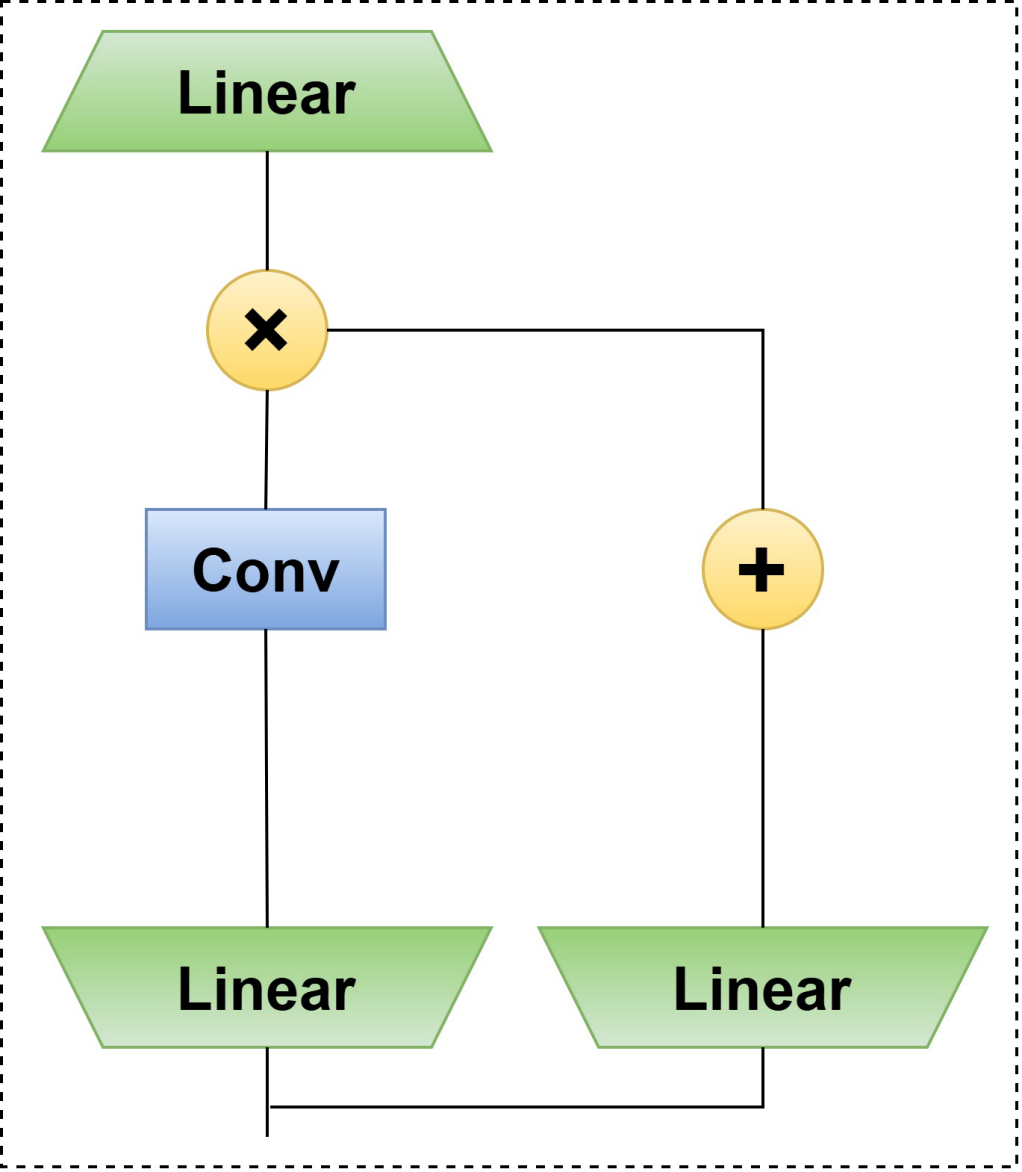
The gated CNN architecture on which the C3k2_Mambaout module is based.

In object detection systems, the design of feature fusion mechanisms decisively impacts a model’s multiscale perception capabilities. Addressing the challenge of identifying minute defect targets (e.g., insect holes) in the Sanhua plum anomaly detection task, this study proposed an innovative C3k2_Mambaout module. By effectively combining deep separable convolutions with gating mechanisms, this module significantly enhances the model’s ability to extract subtle features while maintaining computational efficiency. The overall module architecture is based on the Gated CNN block framework (Yu and Wang, 2024), with its specific structure illustrated in **Figure 6**.

The naming “C3k2_Mambaout” follows a structural convention and acknowledges prior work. “C3k2” indicates a three-layer convolutional block with kernel size 2, emphasizing efficient feature transformation. “Mambaout” draws inspiration from the MambaOut architecture (Yu and Wang, 2024), which questions the necessity of Mamba in vision tasks and adopts a simplified gated CNN design. Thus, the module is fundamentally a gated CNN block tailored for small-defect discrimination, inheriting the dual-path gating mechanism while introducing specific optimizations for Sanhua plum defect detection.

During feature processing, outputs from both paths undergo channel-wise concatenation for information fusion, followed by cross-channel integration. The introduction of a residual connection structure ensures effective gradient propagation, alleviating training difficulties in deep networks.

The performance of the C3k2_Mambaout module was validated on the Sanhua plum fruit defect detection task. Experimental results demonstrated that incorporating this module significantly improved insect-damaged fruit detection precision from the baseline 0.852 to 0.915, proving its effectiveness in enhancing the model’s feature discrimination capability. Through its carefully designed dual-path architecture, the module effectively boosts detection performance for minute defect targets, providing a reliable technical solution for agricultural product quality inspection.

The C3k2_Mambaout module adopts a gated convolution architecture, with its fundamental computation process expressed by Equations 3 and 4:

(3) $$X^{\prime }=Norm(X)$$

(4) $$Y=(TokenMixer(X^{\prime }W_{1})⊙\sigma (X^{\prime }W_{2}))W_{3}+X$$

where TokenMixer(·) represents the feature mixing operation; $W_{1}$, $W_{2}$, and $W_{3}$ denote the learnable parameter matrices; σ is the SiLU activation function; ⊙ indicates element-wise multiplication; and Norm(·) represents normalization processing.

The token mixer TokenMixer(·) in this module is specifically designed with a dual-path parallel architecture to enhance feature extraction capabilities. The first path focuses on the deep extraction of spatial features, constructing multi-level feature representations through cascaded convolutional units. The second path employs a gating mechanism for feature enhancement, generating spatial weights via linear transformations and activation operations on input features to amplify responses in key regions.

### 4.4 Summary of YOLO-CMA collaborative mechanism

The core advantage of the YOLO-CMA architecture lies in the synergistic collaboration of its two enhanced modules. The C2fCIB module plays a pivotal role in the early feature extraction stage, efficiently capturing spatial contextual information through deep separable convolutions to provide a rich feature foundation for subsequent processing. The C3k2_Mambaout module assumes a vital responsibility during the feature fusion stage. It employs a gating mechanism to intelligently filter and enhance features, thereby increasing sensitivity to subtle defects. These two modules complement each other functionally within the feature processing chain, jointly constructing a comprehensive perception system spanning from local details to global semantics.

Regarding the data processing workflow, input images sequentially traverse the four stages of the backbone network to extract multi-scale feature maps. These features undergo fusion through the bidirectional feature pyramid in the neck network, where the C2fCIB and C3k2_Mambaout modules respectively function in the feature extraction and feature integration stages. Finally, the optimized feature maps are fed into the detection head to perform classification confidence prediction and bounding box regression, outputting detection results for five categories of Sanhua plum.

## 5 Experiments and result analysis

### 5.1 Experimental environment

#### 5.1.1 Hardware configuration

Core computing unit: NVIDIA GeForce RTX 3060 GPU with 12GB GDDR6 VRAM, supporting CUDA 12.6 parallel computing architecture. This configuration efficiently handles convolutional operations and gradient backpropagation, meeting training demands for medium-to-large image batches.

#### 5.1.2 Software environment

Development language: Python 3.10.18.

Deep learning framework: Model construction, training, and validation implemented using PyTorch.

Image processing library: OpenCV for image reading, enhancement, and preprocessing.

Support tools: Utilizes thop or torchsummary for model complexity analysis, ensuring reproducibility throughout the experimental process.

#### 5.1.3 Inference speed benchmark setup

To evaluate the real-time capability of the model for edge deployment, inference speed tests were conducted on the same platform (NVIDIA RTX 3060, CUDA 12.6). Testing was performed with a batch size of 1 and an input resolution of 416 × 416. Frames per second (FPS) were recorded as the inference speed metric.

#### 5.1.4 Training parameter configuration

Considering the scale of the Sanhua plum image dataset (10,000 images) and the computational power of the RTX 3060 hardware, this study determined the optimal training parameter combination through multiple rounds of preliminary experiments. The specific configuration is shown in **Table 5**.

Input image size (416 × 416): Effectively controls computational load and GPU memory usage per image while preserving critical defect features like insect holes and cracks. This size aligns with the RTX 3060’s 12GB VRAM and supports efficient training with a batch size of 32.

Batch size (32): Balances gradient update stability and training efficiency. Compared to smaller batches (e.g., 16), a batch size of 32 reduces random fluctuations during training, enhances convergence stability, and prevents memory overflow caused by excessively large batches.

Learning rate and momentum settings: The initial learning rate is set to 0.01, aligning with standard YOLO model conventions to facilitate rapid convergence early in training. The momentum parameter of 0.937 accumulates historical gradient directions to mitigate oscillation during training and enhance parameter update stability.

Training epochs (200): Based on the dataset scale and task complexity, 200 training epochs are set. The first 100 epochs focus on initial model fitting, while the latter 100 epochs refine tuning—preventing underfitting while controlling overfitting risks.

Optimizer selection (auto): Utilizes the YOLO framework’s built-in adaptive optimizer strategy, which automatically selects SGD or AdamW based on the model architecture. This is complemented by a dynamic learning rate decay mechanism, further enhancing training robustness.

Through the above configuration, this study achieved a favorable balance between training efficiency and model performance, providing a stable and reliable training foundation for subsequent module enhancements and model evaluation.

#### 5.1.5 Evaluation metrics

In computer vision, evaluating object detection models requires rigorous quantitative standards. This study employed four core metrics to construct an evaluation framework: Precision (P), Recall (R), Mean Average Precision (mAP), and Average Precision across thresholds (mAP50–95) (Everingham et al., 2010; Hosang et al., 2016). Their definitions and theoretical foundations are as follows.

①Precision (P) measures the confidence of a model’s positive predictions, mathematically expressed as

(5) $$\begin{array}{c} \text{P}=\frac{\text{T}\text{P}}{\text{T}\text{P}+\text{F}\text{P}} \end{array}$$

where TP denotes the number of true positive samples (correctly detected target instances), and FP denotes the number of false-positive samples (incorrectly detected background regions).

②Recall (R): Reflects the proportion of true positive samples correctly predicted by the model, calculated as

(6) $$\begin{array}{c} \text{R}=\frac{\text{T}\text{P}}{\text{T}\text{P}+\text{F}\text{N}} \end{array}$$

where FN is the number of false-negative samples (true targets missed).

③Mean Average Precision (mAP): First, calculate the single-category Average Precision (AP), defined as the area under the Precision–Recall curve (PR curve):

(7) $$\begin{array}{c} \text{A}\text{P}=\int_{0}^{1}\text{P}(\text{R})\text{d}\text{R} \end{array}$$

Then, the mean of the AP values across all classes is taken to obtain mAP:

(8) $$\begin{array}{c} \text{m}\text{A}\text{P}=\frac{\sum_{\text{i}=1}^{\text{N}}\text{A}{\text{P}}_{\text{i}}}{\text{N}} \end{array}$$

where N is the total number of detection classes.

④mAP50-95: Evaluates model robustness by sampling the intersection-over-union (IoU) threshold space. This metric uniformly samples IoU values within the range [0.5, 0.95] at intervals of Δ = 0.05, providing a comprehensive assessment of model performance across varying localization precision requirements.

⑤Parameters (Params): Counted using PyTorch summary tools, measured in M (millions), reflecting model storage requirements.

⑥Computational cost (GFLOPs): Floating-point operations per second for input size 416 × 416, reflecting computational overhead.

⑦Inference speed (FPS): Frames processed per second with batch size = 1 and input size 416 × 416, measured on the test platform to evaluate real-time performance.

### 5.2 Isolated validation of the data augmentation strategy

To quantitatively evaluate the isolated effect of the multi-weather simulation data augmentation strategy, we compared the performance of the YOLOv12 baseline model trained on the original imbalanced dataset versus the augmented balanced dataset under identical experimental settings. The results are presented in **Table 6**.

**Table 6:** Performance comparison of YOLOv12 with and without multi-weather data augmentation.

Model	Category	Precision	Recall	mAP50	mAP50–95
Before data augmentation	All	0.963	0.889	0.918	0.735
	Diseased fruit	0.976	1	0.994	0.993
	Insect-damaged fruit	0.942	0.546	0.648	0.325
	Bird-pecked fruit	0.945	0.915	0.962	0.66
	Cracked fruit	0.957	0.985	0.991	0.705
	Normal fruit	0.996	1	0.995	0.995
After data augmentation	All	0.934	0.889	0.905	0.73
	Diseased fruit	0.969	0.998	0.994	0.974
	Insect-damaged fruit	0.852	0.532	0.612	0.34
	Bird-pecked fruit	0.939	0.921	0.949	0.677
	Cracked fruit	0.94	0.976	0.988	0.714
	Normal fruit	0.979	0.99	0.994	0.946

As shown in **Table 6**, after applying the multi-weather augmentation strategy, the overall mAP50 of the model decreased slightly from 0.918 to 0.905, and the mAP50 for insect-damaged fruit decreased from 0.648 to 0.612. This result shows that, on the standard test set used in this study, the employed data augmentation strategy did not lead to a significant quantitative performance improvement for the baseline model and even introduced minor fluctuations in some metrics, likely due to the simulated environmental noise. Nevertheless, the core value of this strategy lies in the following: 1) thoroughly addressing the class imbalance for critical minority categories like insect-damaged and bird-pecked fruits and 2) potentially enhancing the model’s adaptability to complex environmental variations by simulating diverse weather conditions, which is crucial for its robustness in real orchards with variable lighting and weather. The fluctuation in performance metrics also suggests that future augmentation strategies need to find a better balance between injecting diversity and preserving feature fidelity.

### 5.3 Ablation study analysis

Based on the above analysis, this study proposed targeted module optimization schemes and validated their effectiveness through systematic ablation experiments, with results shown in **Table 7**.

**Table 7:** Ablation experiment design based on YOLOv12 and analysis of each module’s contribution to final performance.

Category	Baseline YOLOv12	C2fCIB	C3k2_Mambaout	Precision	Recall	mAP50	mAP50–95
Diseased fruit	✓	×	×	0.969	**0.998**	0.994	0.974
	✓	✓	×	0.984	0.995	**0.995**	0.972
	✓	×	✓	0.981	0.99	0.994	**0.976**
	✓	✓	✓	**0.988**	0.99	0.993	0.972
Insect-damaged fruit	✓	×	×	0.852	0.532	0.612	0.34
	✓	✓	×	0.856	0.542	0.632	**0.352**
	✓	×	✓	**0.915**	0.496	0.626	0.347
	✓	✓	✓	0.91	**0.545**	**0.639**	0.351
Bird-pecked fruit	✓	×	×	0.939	**0.921**	**0.949**	**0.677**
	✓	✓	×	0.942	**0.921**	**0.949**	**0.677**
	✓	×	✓	**0.951**	0.905	0.947	0.666
	✓	✓	✓	0.941	0.909	**0.949**	0.666
Cracked fruit	✓	×	×	0.94	0.976	0.988	0.714
	✓	✓	×	0.939	0.964	**0.989**	0.722
	✓	×	✓	0.945	**0.978**	**0.989**	**0.724**
	✓	✓	✓	**0.95**	0.976	0.983	0.722
Normal fruit	✓	×	×	0.979	**0.99**	**0.994**	0.946
	✓	✓	×	**0.984**	0.988	**0.994**	0.941
	✓	×	✓	0.977	0.985	**0.994**	0.939
	✓	✓	✓	0.979	0.988	0.993	**0.949**

The bold part indicates the highest value among the four metrics of all models in the corresponding detection category.

In-depth analysis of **Table 7** yields the following key conclusions.

Performance contribution of the C2fCIB module: When the C2fCIB module is introduced independently, the model demonstrates significant improvement in the insect-damaged fruit detection task. Specifically, the mAP50 for insect-damaged fruit increased from the baseline 0.612 to 0.632, while recall rose from 0.532 to 0.542. This indicates that the C2fCIB module enhances the network’s feature extraction capabilities, effectively strengthening the perception and propagation of defect features such as minute insect holes, thereby improving the model’s recall performance.

Performance contribution of the C3k2_Mambaout module: After introducing the C3k2_Mambaout module independently, the model showed an improvement in pest-damaged fruit detection precision, rising significantly from the baseline 0.852 to 0.915. This demonstrates that the module’s unique feature aggregation mechanism enhances the model’s ability to distinguish subtle features, effectively reducing confusion between worm-damaged fruit and other similar features and substantially decreasing false positives.

Module synergy analysis: When C2fCIB and C3k2_Mambaout modules operate synergistically, the model achieves optimal overall performance in insect-damaged fruit detection. At this point, mAP50 reaches its peak value of 0.639, while maintaining a high precision rate of 0.910 and improving recall to 0.545. This synergistic effect clearly demonstrates the complementary functionalities of both modules: the C2fCIB module primarily enhances small object feature extraction capabilities, addressing the “incomplete detection” issue, while the C3k2_Mambaout module focuses on optimizing discriminative feature aggregation, solving the “inaccurate recognition” challenge. Their effective integration enables the improved model to achieve dual enhancements in precision and recall for small object defect detection tasks.

**Figure 7:**
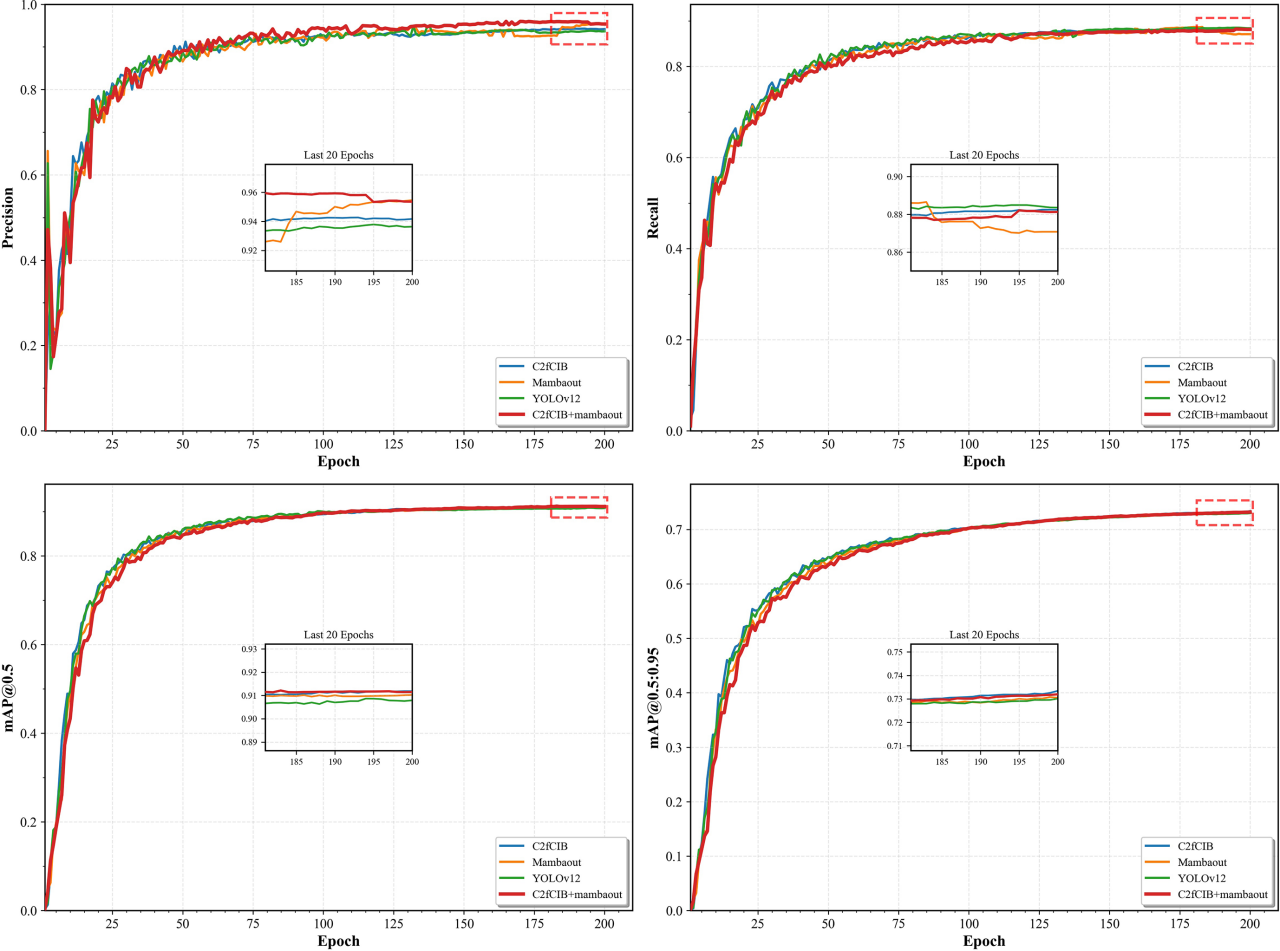
Comparison of various metrics of the ablation study models.

Experimental validation demonstrates the YOLO-CMA model’s outstanding performance in detecting abnormal Sanhua plum fruits. Ablation test results (**Table 5**) reveal that the complete model achieves a mAP50 of 0.639 for insect-damaged fruit detection, with precision rising to 0.910 and recall increasing to 0.545—representing comprehensive performance gains over the baseline model. The contribution of each module is quantified through ablation experiments, with performance comparisons shown in **Figure 7**, visually illustrating the enhancement effect of each improved component on the final performance.

Through a carefully designed combination of modules and feature processing workflows, the YOLO-CMA architecture effectively addresses key technical challenges in detecting abnormal Sanhua plum fruits while maintaining model efficiency. The model’s remarkable performance in detecting small-scale defects like insect-damaged fruits provides a reliable technical solution for agricultural product quality and safety inspection, offering significant practical value and promising application prospects.

### 5.4 Comparative analysis with mainstream YOLO models

To comprehensively evaluate the overall performance of the YOLO-CMA model, this study systematically compared it with that of current mainstream YOLO series models. As shown in **Table 8**, under identical experimental conditions, YOLOv8, YOLOv10, YOLOv11, YOLOv12, and the proposed YOLO-CMA model exhibited distinct performance characteristics in the Sanhua plum abnormal fruit detection task.

**Table 8:** Comprehensive comparison of YOLO-CMA and mainstream YOLO series models in precision and efficiency.

Model	Precision	Recall	mAP50	mAP50–95	GFLOPs	Parameters	FPS
YOLOv8	0.958	0.876	0.91	0.738	6.9	2691183	77.9
YOLOv10	0.937	0.886	0.918	0.726	8.4	2708990	63.1
YOLOv11	0.946	0.892	0.917	0.742	6.4	2590815	60.5
YOLOv12	0.936	0.883	0.907	0.73	6	2520639	36.7
YOLO-CMA	0.954	0.881	0.911	0.732	5.9	2425693	37.9

As shown in **Table 8**, YOLO-CMA achieved an inference speed of 37.9 FPS while maintaining competitive detection accuracy, slightly higher than YOLOv12 (36.7 FPS) but lower than the more computationally intensive YOLOv10 (63.1 FPS) and the fastest model, YOLOv8 (77.9 FPS). Nonetheless, YOLO-CMA exhibited both a lower parameter count and a lower computational cost than these models, demonstrating a superior balance between accuracy and efficiency, making it particularly suitable for resource-constrained real-time edge detection scenarios.

Analyzing precision metrics, all models performed comparably in terms of precision, recall, and mAP. YOLOv8 achieved the highest precision at 0.958, YOLOv11 slightly outperformed others in recall at 0.892, and YOLOv10 attained the optimal mAP50 value of 0.918. The proposed YOLO-CMA model achieved a precision of 0.954, approaching the optimal level, while maintaining a good balance between recall and mAP metrics.

**Figure 8:**
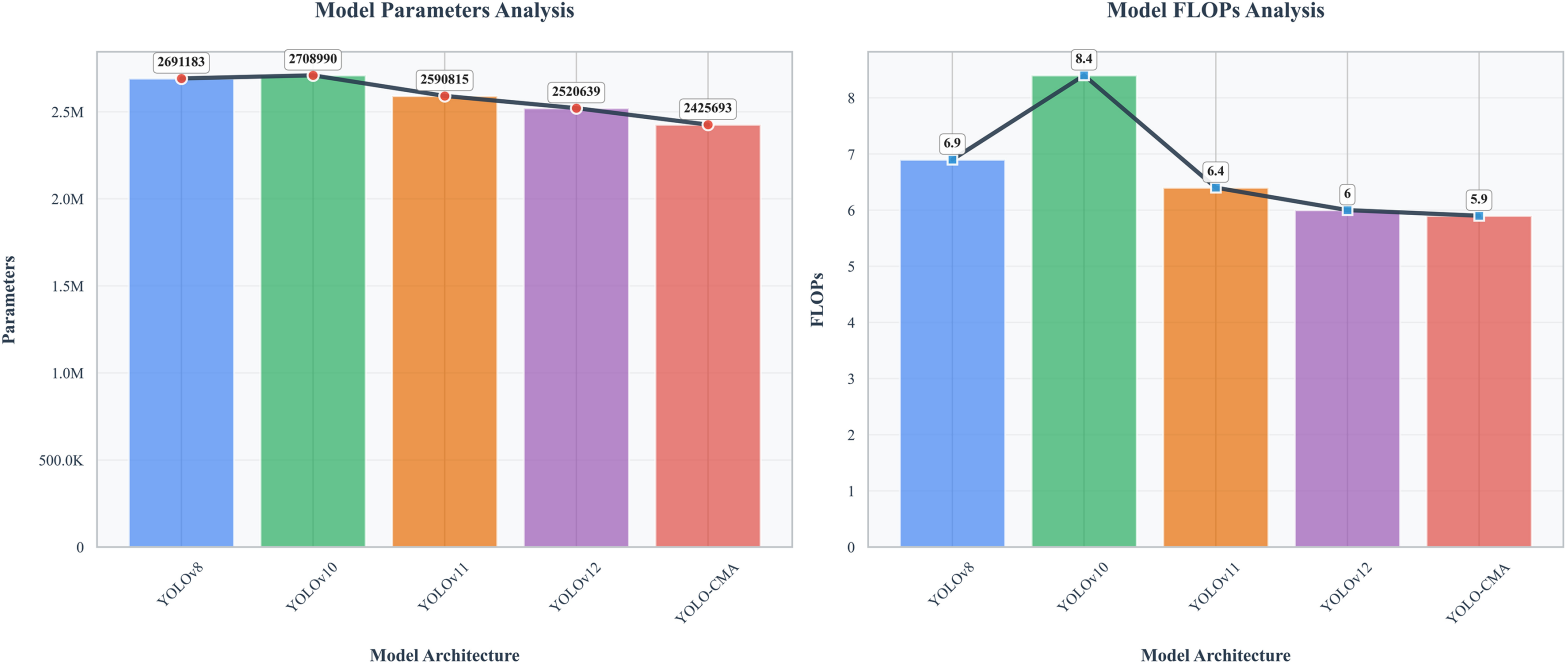
Comparison of computational and parameter amounts of different YOLO versions.

In terms of computational efficiency, YOLO-CMA demonstrated significant advantages. As shown in **Figure 8**, the model required only 5.9G of computational effort and 2.43M parameters, representing the lowest values among all comparison models. Compared to YOLOv10, YOLO-CMA reduced computational complexity by 29.8% and parameter count by 10.3% while maintaining competitiveness in key performance metrics. This efficiency gain primarily stemmed from the optimized design of the C2fCIB and C3k2_Mambaout modules, which achieved efficient feature extraction and fusion through deep separable convolutions and gating mechanisms.

From a comprehensive performance perspective, YOLO-CMA achieved an optimal balance between precision and efficiency. While slightly underperforming certain benchmark models in isolated metrics, it significantly reduced computational complexity and parameter size while maintaining high detection precision. This characteristic makes it particularly suitable for deployment on resource-constrained edge devices, meeting the practical demands of real-time Sanhua plum detection.

Notably, YOLO-CMA’s specialized optimization for detecting small targets like insect-damaged fruits was validated in this comparison. Although its overall mAP50 metric trailed the optimal model by only 0.7 percentage points, its detection capability for minute defects had greater practical value in real-world applications. Furthermore, the model’s efficiency facilitated subsequent engineering deployment and batch processing, demonstrating promising application prospects.

In summary, through innovative module design and architectural optimization, the YOLO-CMA model significantly enhanced computational efficiency while maintaining detection precision, providing a solution that balances performance and practicality for Sanhua plum defect detection. This model holds significant value for promotion and application in the field of agricultural product quality inspection. The experiments demonstrated that YOLO-CMA achieved a high inference speed (37.9 FPS) while maintaining high accuracy, indicating strong potential for edge deployment.

### 5.5 Visualization results and analysis

**Figure 9:**
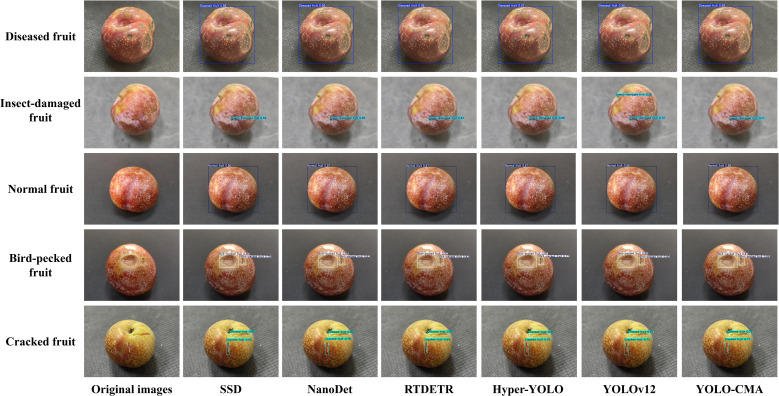
Comparison of visual detection results from various mainstream models (from left to right): original image, SSD, NanoDet, RT-DETR, Hyper-YOLO, YOLOv12, and the proposed YOLO-CMA.

To systematically evaluate the YOLO-CMA model’s performance in real-world detection scenarios, this study conducted an in-depth qualitative analysis by visually comparing the detection results of multiple mainstream detection models—including SSD, NanoDet, RT-DETR, Hyper-YOLO, YOLOv12, and the proposed YOLO-CMA—on the test set. **Figure 9** presents comparisons of multiple representative detection samples covering different defect types, with the panels arranged from left to right, as follows: original image, SSD, NanoDet, RT-DETR, Hyper-YOLO, YOLOv12, and YOLO-CMA.

Detailed analysis of the visual results yielded the following key observations.

In the detection of insect-damaged fruits, the comparative visualization clearly revealed the strengths and weaknesses of each model. SSD and NanoDet tended to under-detect, with generally low confidence scores. RT-DETR and Hyper-YOLO showed higher sensitivity, but the confidence levels across different insect holes were inconsistent. Notably, while the baseline model YOLOv12 detected most insect holes, it exhibited a pronounced false-positive problem; it frequently misclassified natural fruit speckles or texture variations as insect damage, which is evident across multiple sample rows. In contrast, YOLO-CMA maintained a sensitivity similar to that of RT-DETR and Hyper-YOLO but significantly reduced such false alarms, demonstrating a more precise ability to distinguish true defects from background texture. For example, in the second sample row, YOLO-CMA correctly identified the insect hole with a relatively high confidence (0.71) without generating spurious bounding boxes on the surrounding skin, whereas YOLOv12 produced an additional, incorrect bounding box on a similar-looking spot.

Regarding detection stability in complex backgrounds, YOLO-CMA, RT-DETR, and Hyper-YOLO demonstrated superior robustness compared to SSD and NanoDet. Across different samples, YOLO-CMA maintained a high detection recall for diseased and cracked fruits with confidence scores higher than other models.

The visual comparison emphasizes that detecting ultra-small defects (e.g., insect holes close to 1 mm) remains challenging for all models. Typical failure modes include 1) missed detections, especially by SSD and NanoDet for faint, low-contrast holes; and 2) false positives, most notably exhibited by YOLOv12, which confuses natural skin patterns with defects. Although YOLO-CMA effectively mitigates both issues, it is not entirely immune; in some extreme cases, very subtle holes under heavy shadow or highly clustered defects can still be missed or assigned low confidence. These observations highlight the intrinsic difficulty of microscopic defect detection against complex biological textures.

From the perspective of model improvement, these visual advantages stem from the architectural innovations of YOLO-CMA. The C2fCIB module enhances feature reuse efficiency and gradient propagation in shallow layers, improving sensitivity to subtle low-level features. The C3k2_Mambaout module utilizes a gating mechanism to adaptively weight and fuse multi-scale features, enhancing the focus on critical defect regions while suppressing noise. Their synergistic operation enables YOLO-CMA to achieve a better balance between high recall and high precision compared to other models, as visually confirmed.

In summary, the visual analysis qualitatively validates YOLO-CMA’s advantages in detection accuracy and robustness over a range of mainstream detectors (SSD, NanoDet, RT-DETR, Hyper-YOLO, and YOLOv12), particularly in reducing false positives for insect damage while maintaining high confidence. It also honestly reflects the remaining challenges in ultra-small defect detection. These findings corroborate the quantitative evaluations and collectively demonstrate that YOLO-CMA provides a more reliable visual detection solution for minor defects in Sanhua plums, suitable for practical deployment in precision agriculture.

## 6 Discussion

### 6.1 Analysis of model improvement mechanism effectiveness

The proposed YOLO-CMA model significantly enhances detection capabilities for minute defects in Sanhua plums—particularly insect-damaged fruits—by introducing two core modules, C2fCIB and C3k2_Mambaout, building upon the baseline YOLOv12. The C2fCIB module employs a compact inverse-residual structure and depth-separable convolutions to enhance shallow feature extraction while effectively controlling computational complexity, mitigating the dilution of fine-grained features in deep networks. The C3k2_Mambaout module utilizes a gating mechanism and dual-path feature fusion to strengthen attention focus on critical regions, thereby improving the model’s ability to distinguish minute defects. Their synergistic effect is fully validated in ablation experiments: mAP50 for insect-damaged fruit improves from baseline 0.612 to 0.639, precision rises from 0.852 to 0.910, and recall increases from 0.532 to 0.545, demonstrating the rationality and effectiveness of the module design.

### 6.2 Comparison and positioning with existing research

Unlike studies like those on SAW-YOLO (Wu et al., 2024) or CTB-YOLO (Chutichaimaytar et al., 2025) focusing on “generic small objects”, YOLO-CMA specifically tackles the more extreme sub-challenge of “microscopic defect discrimination against complex textures”. While prevailing models often enhance overall architecture, YOLO-CMA adopts a “Collaborative-Decoupled” paradigm: instead of a single, more complex module, it employs two lightweight, specialized modules (C2fCIB and C3k2_Mambaout) to solve the distinct sub-problems of “feature retention” and “discriminative fusion”.

Thus, YOLO-CMA’s advancement lies in its targeted precision and efficiency. As shown in **Table 7**, it achieves significant accuracy gains for challenging categories like insect-damaged fruit at the lowest computational cost, demonstrating that decoupled optimization of key bottlenecks is more effective than holistic architectural strengthening. This offers a new lightweight design insight for specific high-difficulty agricultural inspection tasks.

### 6.3 Contributions and limitations of the data augmentation strategy

The multi-weather simulation data augmentation strategy proposed in this study primarily contributes by systematically addressing the class imbalance issue in Sanhua plum defect detection and providing a robustness training foundation against complex real-orchard scenarios through the simulation of four typical conditions: sunny, rainy, foggy, and nighttime. However, as shown in the controlled experiment in Section 5.2, its quantitative impact on improving the YOLOv12 baseline model’s performance on the standard test set is limited. This clarifies the strategy’s primary role: it acts as a preprocessing and regularization technique designed to enhance model generalization and environmental adaptability, rather than serving as a “silver bullet” for directly improving baseline accuracy. Future work will explore more feature-preserving augmentation methods and directly validate their effectiveness in improving model robustness for real-world deployment on cross-environment test sets.

### 6.4 Limitation analysis

Although YOLO-CMA demonstrates outstanding performance in Sanhua plum defect detection, the following limitations remain.

1. Model generalization requires further validation: Current training and testing rely solely on data from a single production area. Systematic evaluation across different regions and Sanhua plum varieties is pending, necessitating future validation with broader datasets.
2. Detection of extremely small targets remains challenging: While insect-damaged fruit detection performance has significantly improved, the model still exhibits false negatives and false positives for micro-pores smaller than 1 mm in diameter, indicating room for optimization in detecting extremely small targets.
3. Real-time performance and power consumption balance untested on actual edge devices: While the model shows potential for edge deployment in terms of computational load and parameter count, its actual inference speed and power consumption have not been tested on embedded devices (e.g., Jetson Nano and Raspberry Pi). Its practical deployment efficiency requires further evaluation.

## Data Availability

The dataset has been made publicly available at https://github.com/wo-boboya/Sanhua-Plum-Multi-Weather-Enhanced-Dataset.
